# Numerical Proof-of-Concept of Monolithic AC-LGAD Detectors for 4D Particle Tracking

**DOI:** 10.3390/s25134185

**Published:** 2025-07-04

**Authors:** Marco Mandurrino, Manuel Da Rocha Rolo, Angelo Rivetti, Giovanni Margutti, Giuseppe Di Nicolantonio, Lucio Pancheri

**Affiliations:** 1 Istituto Nazionale di Fisica Nucleare Torino, Via Pietro Giuria 1, 10125 Torino, Italy; darochar@to.infn.it (M.D.R.R.); rivetti@to.infn.it (A.R.); 2 LFoundry S.r.l., Via Pacinotti 7, 67051 Avezzano, Italy; giovanni.margutti@lfoundry.com (G.M.); giuseppe.dinicolantonio@lfoundry.com (G.D.N.); 3 Dipartimento di Ingegneria Industriale, University of Trento, Via Sommarive 9, 38123 Trento, Italy; lucio.pancheri@unitn.it; 4 Trento Institute for Fundamental Physics and Applications, Istituto Nazionale di Fisica Nucleare, Via Sommarive 14, 38123 Trento, Italy

**Keywords:** 4D-tracking, AC-LGAD, CMOS, gain layer, LGAD, RSD, silicon detectors, TCAD

## Abstract

We present the numerical proof of a new sensor concept, based on the Resistive AC-Coupled Silicon Detectors (RSDs) paradigm and standard CMOS process, which benefits from having a 100% fill factor and embedded front-end electronics. The compatibility between these two technologies has been investigated, and our encouraging results suggest that this target could be reliably achieved, enabling the possibility to considerably boost the performance of current silicon detectors intended for timing and 4D-tracking.

## 1. Introduction

The need for very precise track reconstruction of charged particles in present and near-future high-energy physics (HEP) experiments requires us to concurrently achieve high resolution of both space and time measurement with the same silicon detector (4D-tracking) [1,2,3,4]. Moreover, in view of dealing with large fluxes of particles and with the constraint of limited space for installation, detectors must have the following properties: efficient, to cope with the increased number of interactions; reliable, to extend their radiation hardness and lifetime; and compact, to achieve fast data acquisition and low materialbudget.

At present, several technologies are under consideration for the development of particle trackers in high-radiation environments like the high-luminosity LHC (HL-LHC) at CERN. The most promising solution in terms of time resolution is the Low-Gain Avalanche Diode (LGAD) technology (∼20 ps) [5]. Thanks to the charge multiplication mechanism, LGADs are characterized by large and fast signals, but so far, even the best performances reported in the literature refer to stand-alone sensors without an integrated front-end [6]. Concerning the spatial reconstruction of tracks, instead, one of the best options is represented by pixel detectors that, in principle, can be produced with collection electrodes as small as required by the experiment. Assuming a binary readout, their space resolution can be calculated as the pixel pitch divided by $\sqrt{12}$. However, high segmentation levels cannot feature the internal gain because the field uniformity required by the LGAD approach is incompatible with small pixels (pitch less than ∼100µm). For this reason, AC-LGADs, an evolution of the standard LGAD technology based on the Resistive AC-Coupled Silicon Detectors (RSDs) concept, have been designed in the past few years [7,8,9]. Thanks to their continuous gain layer throughout the sensor area and the analog readout scheme, RSDs are well known for their 100% fill factor, outstanding spatial resolution (approximately two orders of magnitude lower than the sensor pitch) [10], and timing performances similar to standard LGADs [11].

Due to the tight upgrade timeframe scheduled by most of the HEP experiments, the need to rapidly develop particle detectors for high-precision space reconstruction and time tagging arises. Two approaches are possible: hybrid or full monolithic AC-LGAD integration. However, while the first one allows us to disentangle the sensor complexity from the electronics, at the moment, it is still an expensive technology, and the resulting material budget is beyond the scope of typical HL-LHC scenarios. Other important aspects in the upgrade of most of the present HEP experiments and in the design of detectors for future colliders are as follows: (i) the production costs, (ii) the overall power consumption, and (iii) the material budget, which should be as small as $x/X_{0}≲0.1\%$ (where $X_{0}$ is the material radiation length and *x* its thickness). To optimize all these aspects, a CMOS monolithic integration seems to be the most effective strategy, thanks to relatively low production costs per unit area, the optimized coupling between sensor and readout, which is beneficial in terms of power consumption, and reduced interaction of charged particles with matter compared to the hybrid solution. Moreover, several attempts to reach competitive performances in particle tracking have been already successfully carried out, demonstrating that it is possible to obtain time resolutions lower than 40 ps with a ∼100 µm pitch monolithic sensor, although with a (non-standard) SiGe BiCMOS process [12]. Given all these considerations, here we focus on the second approach, i.e., CMOS monolithic integration.

With the last engineering run (2023) of the ARCADIA project at INFN, we already proved that the LFoundry standard 110 nm CMOS technology node can be properly extended to produce LGADs with embedded front-end electronics by adding to the process flow a *p*-type gain implanted beneath the collection electrode [13,14]. In the present modeling study, we aim to take a step forward in the sensor concept by proposing an innovative detector, designed to concurrently achieve a micron-level spatial resolution and timing properties comparable to standard LGADs. As we are interested in assessing the possibility of producing monolithic AC-LGADs, we designed a numerical study to set up an investigation on the compatibility between the RSD technology and the CMOS process.

## 2. Device Description

**Figure 1:**
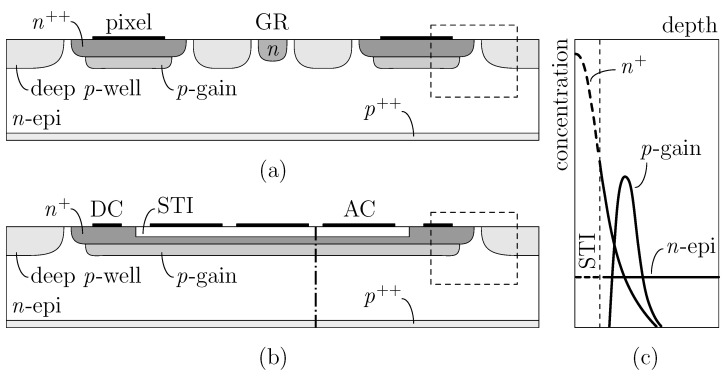
Schematic cross-section of a CMOS-LGAD (**a**) and the CMOS-AC-LGAD device proposed in this work (**b**). Plot (**c**) shows the implant profiles (not to scale) along the dash–dot vertical cutline crossing the CMOS-AC-LGAD.

An example of CMOS-LGAD from the ARCADIA third engineering run (fabricated by LFoundry, Avezzano, Italy) can be found in Figure 1a, where a simplified cross-section of two nearby pixels is sketched. The $n^{++}$ implants correspond to the sensing zones, whereas the electronics is hosted by the surrounding deep *p*-wells. As in the case of LGADs, to achieve an effective electrical isolation between the $n^{++}$ electrodes and, in particular, between the high multiplication fields, some other *p*- and eventually also *n*-type segmentation implants are usually implemented. This additional space devoid of multiplication introduces what is commonly known as a *dead area*, a feature that lowers the geometrical acceptance and, in turn, worsens the tracking efficiency. Moreover, a discontinuous gain compromises the weighting field uniformity and limits the detector timing capabilities.

To overcome the issues arising from a segmented layout and exploit the maximum possible coverage of AC-LGADs, we carried out several TCAD numerical simulations to test a new scenario, where signal formation and propagation are achieved through an RSD scheme (Figure 1b). Here, in place of stand-alone collection regions, a continuous $n^{+}$ electrode and gain layer have been considered. The capacitive component is obtained by introducing in the active area a shallow trench isolation (STI), a feature generally already used in the CMOS process. Unlike traditional AC-LGADs, which have two separate handles—the $n^{+}$ implant, which drives the resistive component, and the dielectric, determining the capacitive coupling—here, the use of trenches leads to a superposition of the two elements.

As reported in Figure 1c, showing the epitaxial, electrode, and gain profiles along the dash–dot cutline in (b), the dielectric acts as a buffer layer for the ion implantation process, modifying the shape of the $n^{+}$ implant with respect to standard AC-LGADs. In the active area, the STI is responsible for the capacitive coupling, and the *n*-type implant behaves as a resistive layer (slowing down the charges enough for a signal to be produced on the AC electrodes). At the sensor periphery, out of the trench, the $n^{+}$ is fully intact, and the electrode works exactly as in a CMOS-LGAD, i.e., collecting multiplied electrons via the DC contact. As pointed out by the dashed boxes in (a) and (b), the same periphery layout has been considered in both devices.

In the following paragraphs, the roles of some important key parameters of the CMOS-AC-LGAD sensor are investigated with the support of a 2D TCAD approach. With regards to device implementation, we had essentially two possibilities: rewrite the process flow to maximize the sensor efficiency, or consider only standard fabrication steps. In line with the purposes of the ARCADIA project, we have chosen to stay consistent as much as possible with the standard CMOS process, without introducing any variation to the technological parameters previously validated in the past productions (see Refs. [13,15]).

## 3. Device Simulation

Since the STI thickness has an impact on both components of the readout RC time constant, the first numerical result we report is a parametric study performed using TCAD Synopsys Sentaurus on the device depicted in Figure 1b. The simulation framework used is based on the drift–diffusion model, with standard values for transport parameters in silicon (see, for reference, [16]). The sensor, built on an *n*-type 50 µm thick epitaxial layer, has three metal pads, with a pitch of 60 μm and a side length of 50 µm, whereas the STI is filled with silicon dioxide. Moreover, a −30 V bias is applied to the backside and 35 V to the top contact. The AC pads are kept at the same potential as the $n^{+}$ cathode, while the *p*-wells hosting the electronics are grounded (0 V). We expect that, by decreasing the trench depth, the capacitive effect becomes stronger, but at the same time, the sheet resistance at the dielectric/silicon interface drops due to the increased $n^{+}$ peak concentration. In contrast, a thick trench weakens the AC coupling and increases the electrode resistivity, suggesting that an optimum trade-off has to be found. To this end, we have chosen two corner cases for the STI height, called thick and thin. They are close to the trench depth generally used in the CMOS platform and represent, respectively, the uppermost and lowermost bounds that still provide good AC coupling (with the thin configuration being approximately 30% lower than the thick one).

**Figure 2:**
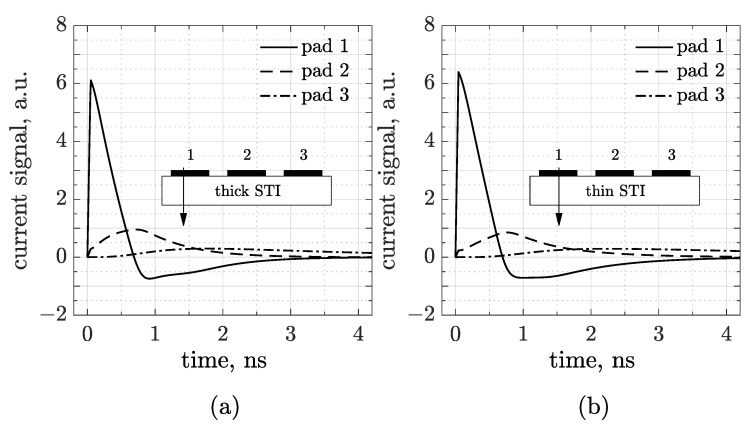
Simulated waveforms in a 3-pad CMOS-AC-LGAD, generated by an MIP crossing the sensor in the center of pad number 1 for a thick (**a**) and (**b**) thin STI configuration. The inset drawings represent a cross-section of the sensor and the arrows show the particle track.

**Figure 3:**
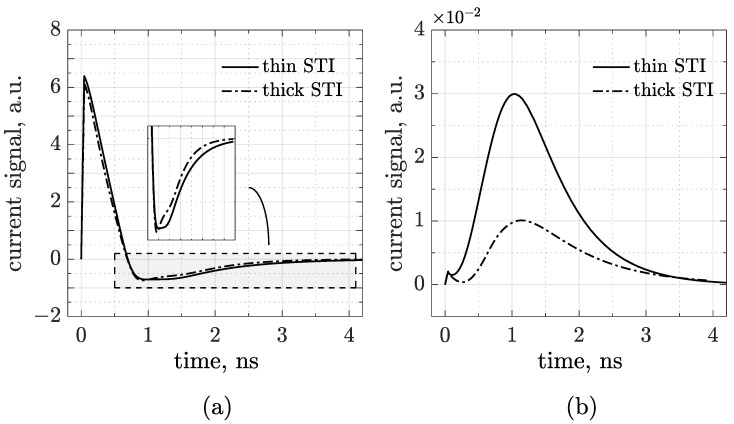
AC (**a**) and DC (**b**) signals acquired, respectively, by pad 1 and the $n^{+}$ electrode in the same simulation of Figure 2. The inset plot on the left shows a zoomed-in view of the discharge lobes enclosed in the gray box.

The waveforms plotted in Figure 2 have been obtained through a transient simulation in which a minimum ionizing charged particle (MIP) is injected on top of the sensor, perpendicularly to the frontside, in the middle of pad number 1 (see the arrow in the inset drawing). The left plot refers to the thick trench and the other to the thin one. The first observation is that AC pulses show a prompt, almost immediate, response. As in traditional AC-LGADs, signals are bipolar with a first dominant peak and a second opposite lobe restoring the total charge to zero. The first lobe arises from the rapid induction of charges generated by ionization as they drift toward the cathode and induce a signal on the AC pads. As these charges reach the resistive layer, they begin to move toward the DC contacts, giving rise to the second lobe. In contrast to standard (hybrid) devices, here the main peak has a shorter rise time, and the amplitude of the discharge process is less pronounced [7]. This seems to indicate strong performances in terms of timing and 4D-tracking, and is probably due to the different technological conditions (e.g., substrate, dielectric, and resistive layer). However, TCAD is not the most suitable tool for making predictions about space and time resolution, while Monte Carlo simulations would be more appropriate for confirming the conclusions drawn from the waveforms presented here. Nevertheless, the most typical feature of having delayed and decreasing signals as the distance from the hit point increases is still preserved. This aspect is fundamental for hit point reconstruction and, in turn, for the determination of the time resolution. Overall, therefore, Figure 2 demonstrates—at least numerically—that it is possible to achieve an RSD readout scheme in the framework of a CMOS sensor, and that the output signals are suitable for both timing and tracking applications. The difference induced by the STI height, anyway, is quite limited. We may notice, for instance, the enhancement of the coupling strength through a slight increase in the main lobe amplitude from the thick to the thin layout. Regarding the discharge, it can be seen that, for the thin STI, the tail is slightly longer than for the thicker one. This is better represented in Figure 3a, where the waveforms acquired by just pad 1 are compared (see a zoomed-in view in the inset).

To investigate the origin behind the counterintuitive trend of the discharge time with the trench depth, we decoupled the two terms contributing to the time constant by checking, for both STI options, the DC signals collected at the $n^{+}$ electrode, where only the resistive component survives (see Figure 3b). It is worth noting that AC pulses are higher and faster than DC signals, with remarkably lower rise time compared to standard CMOS-LGAD. As expected, Figure 3b also indicates that a thick trench induces a smaller and delayed peak with respect to the thinner one, and the baseline recovery is consequently longer (so that the integrated charge is conserved). In summary, at the periphery, a thick dielectric implies a low $n^{+}$ peak concentration, high resistivity and, contrary to what occurs for bipolar signals, a long signal tail.

This allows us to conclude that in the sensor’s active region, the STI thickness mainly determines the coupling capacitance—which dominates the RC time constant—and only marginally affects the electrode resistivity.

**Figure 4:**
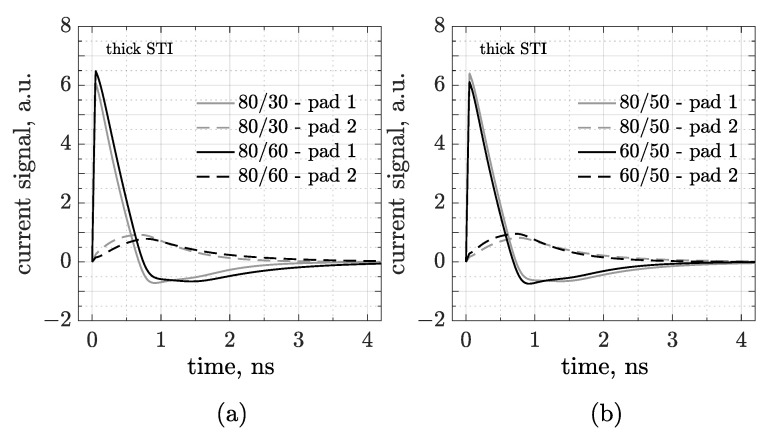
Simulated signals in 3-pad CMOS-AC-LGAD sensors with the same pitch (80 µm) and different pad sizes (**a**) or the same pad size (50 µm) and different pitches (**b**). The particle is injected in the center of pad 1, and signals are acquired by both pad 1 and 2.

Since the readout capacitance is crucial for the operation of our device, another important point to check is whether, and eventually how, the sensor geometry affects the coupling. In the following simulations, we tested different configurations of pitch and pad size (only for the thick STI layout). Figure 4a shows the waveforms acquired by pad 1 and 2 in two 80 µm pitch CMOS-AC-LGADs for an MIP perpendicularly crossing the center of pad 1. The gray curves refer to a 30 μm pad structure, whereas the black ones refer to a doubled pad size. As expected, the coupling—and, in turn, the signal amplitude—is enhanced for larger pads. As reported in Figure 4b, we also investigated the role of pitch in the signal formation simulating two structures with a 50 µm pad size: an 80 (in gray) and a 60 µm pitch (black). As a first observation, neither sensor pitch nor pad size affects the rise time of the main lobe. Secondly, the signal amplitude in pad 1 decreases from the 80/50 to the 60/50 device, whereas in pad 2, it slightly increases, probably because interpad and coupling capacitances are competitive, and this effect is greater if the distance between pads is small.

The last numerical test we report concerns the geometrical efficiency of the sensor. As described in the first paragraphs, the RSD paradigm is known for its 100% fill factor, a consequence of having continuous *p*-gain and resistive implants. Although AC-LGADs are composed of a single uninterrupted diode, the charge-sharing mechanism may lead to a deterioration of the efficiency between pads, making full acceptance a non-straightforward figure of merit.

**Figure 5:**
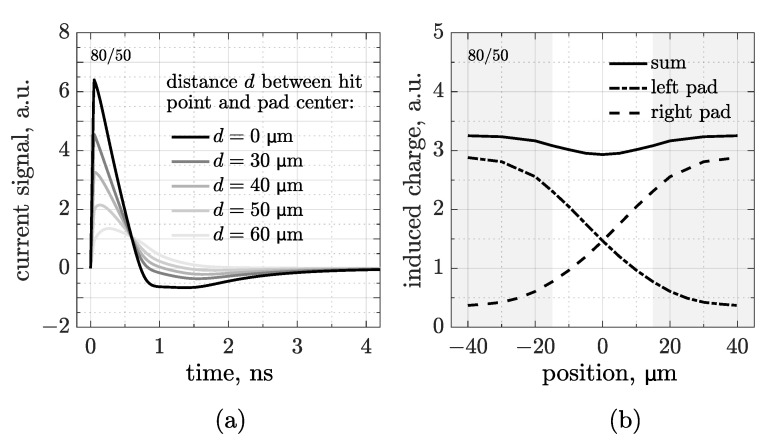
Set of waveforms (**a**) and induced charge (**b**)—calculated as the integral of the first lobe of the signal—simulated during a scan of a particle moving in the region between the centers of two adjacent pads in a 80 µm pitch and 50 µm pad CMOS-AC-LGAD sensor.

So far, we have simulated only MIPs impinging on the center of pads. Therefore, to check the signal properties in the interpad region, a scan was carried out by moving the particle hit point in the space between the centers of two adjacent pads. Figure 5a shows the waveforms simulated at five different positions of this scan in a 80 µm pitch and 50 µm pad CMOS-AC-LGAD, where *d* is the distance between the hit point and the center of the pad that reads the signal. This means that the waveform simulated at d=60µm is generated by a particle crossing the sensor 20µm from the center of the neighboring pad.

As previously observed, the signals show a decreasing amplitude as the particle moves away from the pad. To gain a better understanding of this phenomenon, the integrals of the first lobe acquired by each of the two pads have been plotted over the course of the scan, as shown in Figure 5b. Areas in gray represent the space covered by metal pads. We obtained two symmetrical S-curves (dashed and dot-dashed), whose sum (solid) shows a fairly good uniformity of the induced charge, with only a small fraction of lost information in the middle of the interpad. This trend should enable very precise tracking, for what concerns both the hit point reconstruction and the time-of-arrival calculation.

**Figure 6:**
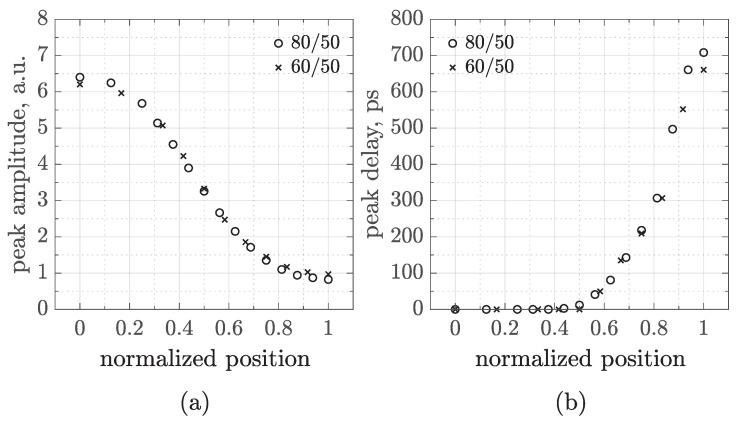
Peak amplitude (**a**) and delay (**b**) as a function of the normalized hit position, obtained by simulating an MIP scan in two 50 µm pad CMOS-AC-LGADs with 80 µm (open circles) and 60 µm (crosses) pitch readout geometries. The position is normalized to account for the different pitches, where 0 and 1 correspond to the centers of two neighboring pads.

Further investigations about the charge sharing are reported in Figure 6. To test the impact of the readout segmentation on signal spreading, two 50 μm pad CMOS-AC-LGADs with different pitch sizes were simulated and compared. Again, an MIP was injected perpendicularly to the sensor surface, moving between the centers of two neighboring pads. Then, the peak amplitude—Figure 6a—and time—Figure 6b—have been plotted as a function of the hit position. Since we simulated two different sensor geometries, the positions have been normalized in such a way that 0 always corresponds to the center of the left pad and 1 to the center of the right pad.

Figure 6 demonstrates that, whatever the sensor geometry, signals spread following the same laws, at least for the values of the pitch here considered and for the figures of merit we focused our attention on, i.e., the peak amplitude and the signal delay, defined as the time difference between peaks (referred to the first lobe of the signals) evaluated during the scan. These plots represent a building block in the design, characterization, and optimization of sensors, as they can serve as a benchmarking tool for interpreting measurements and calibrating reconstruction algorithms.

## 4. Conclusions

We presented a numerical study designed to test the compatibility between the RSD layout and the standard CMOS process, proposing monolithic AC-LGADs as enabling detectors for high-performance 4D-tracking. Several sensor prototypes have been simulated using STI as a capacitive readout element. In the meantime, trenches also modulate the silicon/oxide interface sheet resistance, which sets the resistive component of the RC time constant. First, our simulations proved that RSDs can be proficiently integrated in a CMOS sensor. Then, we observed, by comparing DC and AC pulses, that the signal formation is mainly driven by the coupling effect. This is most likely the reason why the AC pulses are drastically higher and faster than the waveforms obtained via charge collection, a beneficial aspect for timing and, more generally, 4D-tracking applications. We also studied the role of pad pitch and size, observing that the proposed device behaves like traditional AC-LGADs, where the interpad capacitance shapes the coupling, and signal amplitude scales with pad size. Finally, the geometrical efficiency, interpreted as the sensor acceptance, was investigated. By simulating a particle scan between two nearby metal pads, it was observed that there was no significant information loss, in terms of induced charge, in the interpad region. This strongly indicates that the CMOS-AC-LGAD sensor can achieve a real 100% fill factor. Through the analysis of the waveforms obtained in the scan simulation, it was also possible to characterize different sensor geometries from the standpoint of signal propagation and charge sharing. As shown by simulations, signals decrease in amplitude and spread in time as the particle moves far away from the readout pad. And the trend of these figures of merit as a function of the hit position appears to be independent of the sensor geometry, at least for the values of pad pitch and technological parameters considered in the present study.

## Data Availability

Data is contained within the article.

## Abbreviations

The following abbreviations are used in this manuscript:

RSD	Resistive AC-Coupled Silicon Detector
HEP	High-Energy Physics
HL-LHC	High-Luminosity Large Hadron Collider

LGAD	Low-Gain Avalanche Diode
CMOS	Complementary Metal-Oxide-Semiconductor
BiCMOS	Bipolar CMOS
ARCADIA	Advanced Readout CMOS Architectures with Depleted Integrated sensor Arrays
TCAD	Technology Computer-Aided Design
STI	Shallow Trench Isolation
GR	Guard-Ring
MIP	Minimum Ionizing Particle
